# Chemistry Meets Plasmon Polaritons and Cavity Photons: A Perspective from Macroscopic Quantum Electrodynamics

**DOI:** 10.1021/acs.jpclett.4c03439

**Published:** 2025-02-05

**Authors:** Liang-Yan Hsu

**Affiliations:** † Institute of Atomic and Molecular Sciences, Academia Sinica, Taipei 106, Taiwan; ‡ Department of Chemistry, National Taiwan University, Taipei 106, Taiwan; ¶ Physics Division, National Center for Theoretical Sciences, Taipei 106, Taiwan

## Abstract

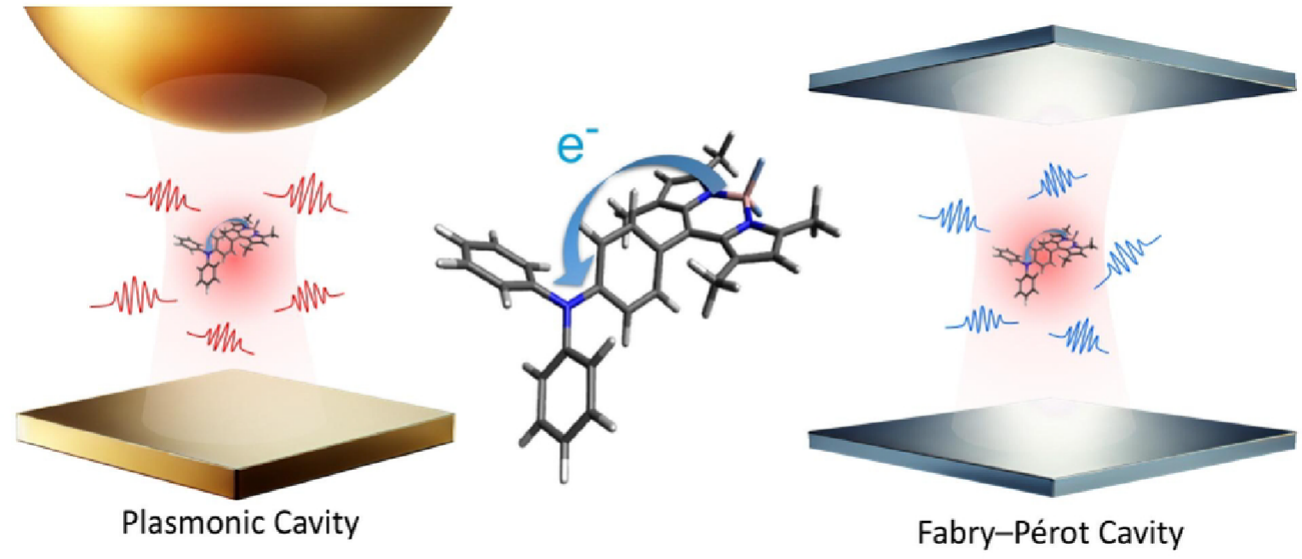

The interaction between light and molecules under quantum electrodynamics (QED) has long been less emphasized in physical chemistry, as semiclassical theories have dominated due to their relative simplicity. Recent experimental advances in polariton chemistry highlight the need for a theoretical framework that transcends traditional cavity QED and molecular QED models. Macroscopic QED is presented as a unified framework that seamlessly incorporates infinite photonic modes and dielectric environments, enabling applications to systems involving plasmon polaritons and cavity photons. This Perspective demonstrates the applicability of macroscopic QED to chemical phenomena through breakthroughs in molecular fluorescence, resonance energy transfer, and electron transfer. The macroscopic QED framework not only resolves the limitations of classical theories in physical chemistry but also achieves parameter-free predictions of experimental results, bridging quantum optics and material science. By addressing theoretical bottlenecks and unveiling new mechanisms, macroscopic QED establishes itself as an indispensable tool for studying QED effects on chemical systems.

The interaction between light and molecules is one of the most fundamental and significant topics in the field of chemistry. Light can be used not only to identify molecules but also to drive photochemical reactions, such as photosynthesis^1−4^ and ozone photolysis.^5−8^ The foundational studies of photochemistry can be traced back to the 19th century with Grotthuss and Draper,^9,10^ while Stark and Einstein established the fundamental laws of photochemistry in the early 20th century.^11^ In spectroscopy, Coblentz made breakthroughs in infrared spectroscopy for analyzing molecular functional groups and chemical bonds,^12^ Raman discovered the Raman effect in molecular scattering spectra,^13^ and Herzberg conducted his pioneering work on molecular vibration and rotation spectra, laying the foundation for modern molecular spectroscopy.^14^ After 1960, with advances in laser technology and theoretical tools, Franken discovered second harmonic generation,^15^ while Bloembergen,^16^ Shen,^17^ and Mukamel^18^ further developed nonlinear optical theory, exploring molecular nonlinear polarizabilities, molecular structures, and vibrational dynamics, with applications in surface chemistry and dynamics studies. During this period, Zewail pioneered the use of femtosecond laser technology to study chemical reaction dynamics and mechanisms,^19^ and attosecond spectroscopy emerged more recently as a powerful tool for probing ultrafast electronic dynamics.^20^ Around the same time, Van Duyne and Schatz utilized surface-enhanced Raman spectroscopy to enhance molecular vibrational signals and detection sensitivity, advancing nanophotonics and plasmonics in the field of chemistry.^21^ In the field of coherent control, Rice, Brumer, and Shapiro laid the theoretical groundwork for quantum control,^22,23^ which was further advanced experimentally by Gerber and Rabitz using femtosecond lasers to control chemical reaction products.^24,25^ Over the past century, physical chemists have used quantum mechanics combined with classical electromagnetic fields to quantitatively describe most interactions between light and molecules. However, while only a few phenomena require the framework of quantum electrodynamics (QED), it is important to note that photons, which underpin all spectroscopy and interactions between molecules, and the effects of both vacuum fluctuations and populated radiation states are direct consequences of QED.^26−28^

Richard Feynman once stated,^29^ ”The theory behind chemistry is quantum electrodynamics.” QED effects, such as the Lamb shift,^30^ have long been considered very small effects in chemistry, but they demonstrate the profound impact of electromagnetic vacuum fields on chemical systems. Spontaneous emission (e.g., molecular fluorescence) and London dispersion forces (a component of van der Waals forces) are examples of QED effects that reveal the quantum nature of electromagnetic fields in chemistry.^31,32^ Spontaneous emission is driven by zero-point fluctuations of the electromagnetic field, facilitating the transition of molecules from excited states to ground states. This process has been widely applied in fluorescence labeling,^33,34^ biosensing,^35,36^ organic light-emitting diodes (OLEDs),^37−39^ and photovoltaic technologies.^40−42^ London dispersion forces arise from the fluctuation of electronic motion and stabilize molecular crystal structures through the formation of instantaneous dipoles, significantly influencing protein tertiary structures^43^ and supramolecular assemblies.^44,45^ QED effects provide chemistry with a new perspective, revealing the critical role of the quantum nature of electromagnetic fields in molecular behavior. However, applying QED effects to control chemical reactions has remained a challenging goal, primarily due to the difficulty of effectively amplifying the impact of electromagnetic vacuum fields (or fluctuations) on chemical processes.

Traditionally, photochemical reactions have been considered to require external light sources to be driven. However, in recent years, studies by Ebbesen and others have challenged this conventional view.^46,47^ By tuning the mirror spacing of a Fabry-Pérot (FP) cavity to adjust cavity photon frequencies, they demonstrated that cavity photons generated by electromagnetic vacuum fluctuations can resonate with the electronic transitions and vibrational frequencies of molecules without an external light source, achieving electronic strong coupling^48^ and vibrational strong coupling,^46^ respectively. These studies not only controlled the branching ratio of products in chemical reactions^49^ but also successfully utilized the cavity to modulate chemical reaction rates,^50^ promoting the rise of the field of polariton chemistry (cavity chemistry or QED chemistry). In addition to Ebbesen’s experimental work, Simpkins et al. also demonstrated the feasibility of using cavities to control chemical reaction rates and provided possible mechanistic explanations.^51^ Xiong et al. showed that cavities influence intermolecular vibrational energy transfer and intramolecular vibrational redistribution,^52,53^ while Delor et al. utilized cavity photons to form exciton-polaritons, thereby affecting exciton diffusion coefficients.^54^ To date, most experimental studies have been designed based on FP cavity systems, but vacuum-field-driven chemical reactions are not limited to FP cavity systems and can also be realized in other systems. For example, Verdelli, Wei, and Gomez Rivas used surface lattice resonance and vibrational strong coupling of C=O stretching within nonlocal metasurfaces to accelerate the reaction rate of p-nitrophenyl acetate without an external light source.^55^ These experimental studies not only reveal the feasibility of using quantum vacuum fields to control chemical reactions but also open up new directions for exploring photochemical reactions based on strong coupling, highlighting the great potential of QED effects in controlling the rate and selectivity of chemical reactions.

With the rapid experimental advancements in polariton chemistry, many theoretical studies have also emerged to understand the nature of strong coupling phenomena. Rubio and collaborators developed the cavity Born–Oppenheimer (BO) approximation^56^ and extended electronic structure methods, such as density functional theory^57^ and traditional quantum chemistry,^58^ to the framework of cavity quantum electrodynamics (cavity QED), revealing how light–matter interactions modify potential energy surfaces.^59^ Herrera and Spano proposed that cavity photons can effectively decouple the collective electronic and nuclear degrees of freedom in molecules, resulting in collective effects in electron transfer.^60^ Feist and collaborators suggested that under strong coupling between light and molecules, the concept of many-molecule reactions triggered by a single photon challenges the second law of photochemistry (Einstein-Stark Law).^61^ Yuen-Zhou and Huo developed a series of theoretical works, including electron transfer theory and transition-state theory, providing crucial insights into chemical reactions under vibrational strong coupling.^62,63^

Despite offering novel perspectives on light–matter interactions under strong coupling, the current framework, such as cavity QED and single-photonic-mode model, faces challenges in experimental design and prediction. First, the strength of light–matter coupling is determined by the free parameter, effective volume, and the cavity loss is another parameter which needs to be experimentally measured, limiting theoretical predictive power for strong coupling conditions.^64^ Second, cavity QED and single-photonic-mode models neglect the influence of infinite photonic modes, which introduces inaccuracies in the quantitative description of intermolecular forces (dispersion forces) and collective effects.^65,66^ Third, cavity QED cannot quantitatively account for how experimental setups (e.g., cavity structure and materials) impact light–matter interactions, restricting its applicability.^67,68^ These challenges highlight the limitations of the current theoretical frameworks, such as cavity QED and single-photonic-mode models, in describing strong coupling phenomena and light–matter interactions in complex environments. To address these challenges, macroscopic quantum electrodynamics (macroscopic QED), developed by Welsch, Scheel, Buhmann et al.,^69−72^ is presented in this Perspective as a more general and comprehensive theoretical framework. By incorporating infinite photonic modes and dielectric effects, macroscopic QED not only addresses the shortcomings of existing models but also provides a powerful tool for studying QED effects on chemical systems. Building on this framework, my group has applied macroscopic QED to investigate molecular fluorescence, resonance energy transfer, electron transfer, nonadiabatic QED effects, and superradiance, offering novel insights and addressing theoretical bottlenecks in polariton chemistry, while leaving room for future applications in broader contexts of quantum optics and nano-optics.

## Macroscopic Quantum Electrodynamics

**Figure 1:**
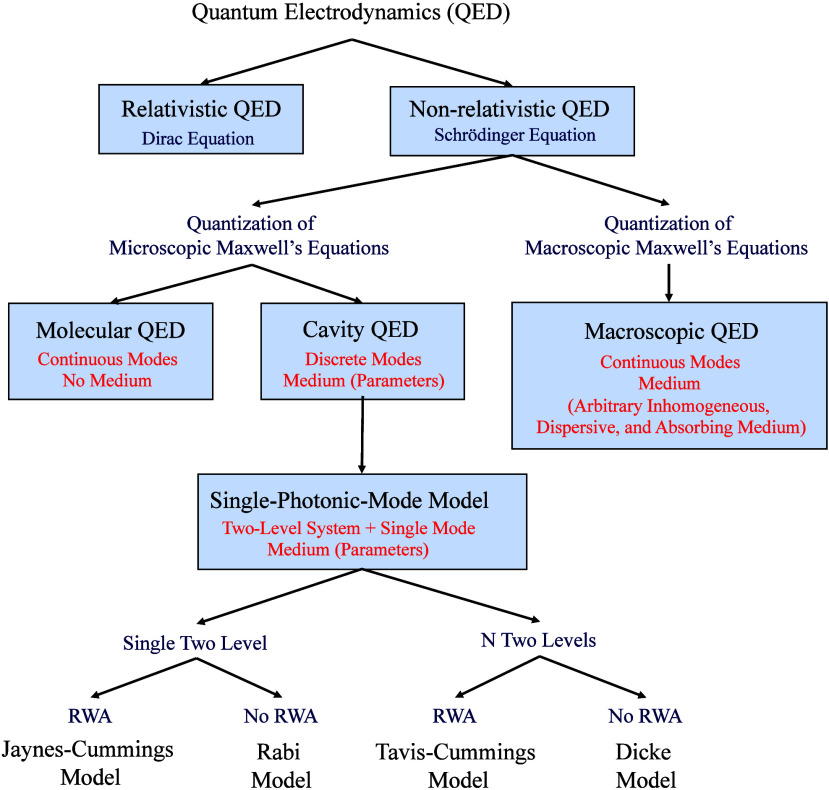
Overview of nonrelativistic quantum electrodynamics. Here RWA indicates rotating-wave approximation.

Before introducing macroscopic QED, I first outline the key differences between macroscopic QED and existing theories in the field of polariton chemistry, as shown in Figure 1. QED can be classified into relativistic QED and nonrelativistic QED, depending on whether the particles are moving at speeds close to the speed of light.^73^ Relativistic QED relies on the Dirac equation and the quantization of the electromagnetic field, focusing on quantum phenomena associated with relativistic effects. On the other hand, nonrelativistic QED is based on the Schrödinger equation and the quantization of the electromagnetic field, emphasizing interactions between quantized electromagnetic fields and matter. In polariton chemistry, most studies focus on the effects of electromagnetic vacuum fluctuations (self-energy effects, radiative processes, and light–matter hybrid states), while typically neglecting the roles of antimatter and spin–orbit coupling in chemical systems.^74^ Under this premise, nonrelativistic QED suffices.

Nonrelativistic QED can be categorized into molecular QED, which is based on the microscopic Maxwell’s equations,^26−28,75^ and macroscopic QED, which is based on the macroscopic Maxwell’s equations.^76^ Molecular QED describes the influence of infinite continuous photonic modes on molecules, where mechanisms such as spontaneous emission, resonance energy transfer, and London dispersion forces can be derived within this framework.^27,77−81^ However, it does not account for the effects of dielectric materials on the quantization of electromagnetic fields. Compared to molecular QED, cavity QED quantizes the microscopic Maxwell’s equations within a confined space, yielding infinite but discrete photonic modes and finding broad applications in quantum optics.^82^ Cavity QED can be further simplified. Suppose we consider a system with only a two-level system and a single photonic mode. In that case, one can derive the Jaynes-Cummings (JC) model,^83^ Tavis-Cummings (TC) model,^84^ Rabi model,^85^ and Dicke model,^86^ collectively referred to as single-photonic-mode models. The differences among these models lie in the number of two-level systems involved: the JC model and Rabi model consider a single two-level system coupled to one photonic mode, while the TC model and Dicke model describe *N* two-level systems coupled to one photonic mode. From a mathematical perspective, the JC model and TC model are obtained under the rotating wave approximation, excluding counter-rotating interactions, whereas the Rabi model and Dicke model include counter-rotating interactions. Within the framework of these four models, the focus is placed on the effect of a single photonic mode on physical or chemical systems, significantly reducing the modeling complexity. Most studies in polariton chemistry adopt cavity QED or single-photonic-mode models as their theoretical foundation.^87−91^

Compared to molecular QED and cavity QED, macroscopic QED not only accounts for the influence of infinite continuous photonic modes on molecules but also describes the effect of dielectric media on these photonic modes. The theoretical development of macroscopic QED can be traced back to the 1940s, when Jauch and Watson proposed phenomenological quantum electrodynamics (Phenomenological QED), which quantized the macroscopic Maxwell’s equations.^92^ However, their approach could not address the dispersion and dissipation of the media or connect to microscopic models. In 1958, Hopfield developed a microscopic model to describe the coupling between electromagnetic fields and lattice vibrations, which yielded polaritons through quantization,^93^ although this work did not establish a connection to the quantization of macroscopic Maxwell’s equations. Subsequently, Hillery and Drummond made advancements in macroscopic QED by incorporating noise^94^ and nonlinear effects.^95,96^ In the physical chemistry community, Knoester, Mukamel, Juzeliunas, and Andrews successively explored spontaneous decay, superradiance, and resonance energy transfer in dielectric media using a Green’s function approach.^97,98^ A critical step in this field was achieved in 1992 by Huttner and Barnett, who introduced a microscopic model for medium-field coupling (commonly referred to as the Huttner-Barnett model).^99,100^ In this model, they treated the media as quantum harmonic oscillators and incorporated Langevin noise operators into macroscopic Maxwell’s equations to quantize the electromagnetic field interacting with absorbing media. Their approach was the first rigorous quantization model capable of handling dissipative and dispersive media, laying the foundation for modern macroscopic QED. Inspired by Huttner and Barnett, the Welsch group further combined Langevin noise operators with dielectric functions using the Kramers–Kronig relation and expressed the quantized electromagnetic field using dyadic Green’s functions.^101^ This advancement extended the framework of macroscopic QED to three-dimensional inhomogeneous, dispersive, and absorbing media.^76^ Under the framework of perturbation theory, the Welsch group also investigated spontaneous emission in dielectric environments, successfully explaining how such environments influence spontaneous emission within the macroscopic QED framework.^102,103^ Buhmann expanded macroscopic QED applications to include studies of dispersion forces, such as the Casimir and Casimir-Polder forces.^104^ Today, macroscopic QED has become an essential theoretical tool in quantum optics and nanophotonics, and a few recent studies have begun exploring its integration with polariton chemistry.

To help readers quickly understand the main discrepancy between macroscopic QED and the mainstream theoretical approaches in polariton chemistry, such as cavity QED, I will first briefly explain how the electric field operators differ between cavity QED and macroscopic QED under the Coulomb gauge. This comparison emphasizes macroscopic QED’s ability to incorporate dielectric environments and infinite photonic modes, extending beyond the constraints of cavity QED. Different gauges apply to different conditions, and for a detailed discussion on gauges, readers can refer to the previous work done by Huo,^105^ Andrews,^106^ and Nazir.^107^ Mathematically, the key distinction between classical electrodynamics and QED lies in their treatment of the electric field: in classical electrodynamics, it is a function, while in QED, it is an operator. In both cavity and macroscopic QED, the electric field operator **Ê**(**r**) at the position **r** can be expressed as the sum of its positive and negative frequency components:
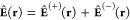
1where **Ê**^(+)^(**r**) represents the positive frequency component and **Ê**^(−)^(**r**) represents the negative frequency component. **Ê**^(+)^(**r**) and **Ê**^(−)^(**r**) are Hermitian conjugates of each other, so evidently the electric field operator **Ê**(**r**) is a Hermitian operator.

In cavity QED, the positive frequency component of the electric field operator is written as
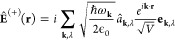
2The positive frequency component of the electric field operator consists of four parts: the prefactor, the photon annihilation operator, the mode function, and the polarization vector. The prefactor includes the photon frequency ω_**k**_ and the vacuum permittivity ϵ_0_. The photon annihilation operator *â*_**k**, λ_ is associated with the wave vector **k** and polarization λ, describing the annihilation of photons. The mode function *e*^*i* **k** ·**r**^/√*V* describes the spatial distribution of photons, where *V* represents the mode volume as a free parameter. Under free-space boundary conditions, it takes the form of a plane wave *e*^*i* **k** ·**r**^. In the Coulomb gauge, the polarization vectors **e**_**k**, λ_ correspond to the directions of two transverse modes of electromagnetic waves, which are orthogonal to the wave vector **k**.

In macroscopic QED, the positive frequency component of the electric field operator is expressed as follows,^108^
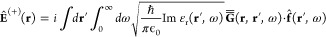
3The positive frequency component of the electric field operator consists of three parts: the prefactor, the dyadic Green’s function, and the Bosonic vector field. The prefactor  reflects the energy absorption of the medium through the imaginary part of the permittivity ε_r_(**r**′, ω). The dyadic Green’s function  satisfies the macroscopic Maxwell equations,^109,110^
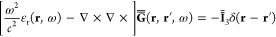
4where  is a 3 × 3 identity matrix, and δ(**r**–**r**′) is the three-dimensional Dirac delta function. The dyadic Green’s function from classical electrodynamics captures how electromagnetic fields propagate in complex dielectric environments, accounting for absorption, dispersion, and boundary effects. The bosonic vector field **f̂**(**r**, ω) is the quantized field operator corresponding to the annihilation operators of the electromagnetic field. The field density, **f̂**^†^(**r**, ω) **f̂**(**r**, ω), contributes to the total field energy *H*_Field_=∫*d***r** ∫_0_^∞^*dω* ℏω **f̂**^†^(**r**, ω) **f̂**(**r**, ω). Note that in macroscopic QED, the bosonic vector field **f̂**(**r**, ω) is derived from the quantization of medium excitations and photons, making them inherently linked to polaritons.^76^ In macroscopic QED, polarization is inherently embedded in the dyadic Green’s functions and the bosonic vector field.

By comparing the positive frequency components of the electric field operators in eqs 2 and 3, it is evident that macroscopic QED leverages the dyadic Green’s function and the bosonic vector field to accurately account for absorption, dispersion, and geometric boundary conditions in dielectric environments. It also provides a more quantitative description of interactions between molecules and cavity photons or plasmon polaritons. While molecular QED is more suited to describing molecule-photon interactions in free space, the macroscopic QED framework expands the scope of interpretation and has the potential to overcome the theoretical limitations of cavity QED and single-mode models, which inadequately account for infinite photonic modes and dielectric effects. It should be noted that, unlike relativistic QED and molecular QED, macroscopic QED is not an exact theory due to the lack of an exact quantum description of a medium.^111^ Nevertheless, with advances in electronic structure calculations, dielectric functions can be obtained from first-principles calculations under certain approximations. As a result, macroscopic QED can be viewed as an effective or coarse-grained framework for describing photonic baths. In the next section, I will discuss recent advancements by my group, focusing on the application of macroscopic QED to plasmonic materials and cavity-enhanced systems, highlighting their potential in chemical and quantum photonic systems.

## Macroscopic QED in Chemistry

**Figure 2:**
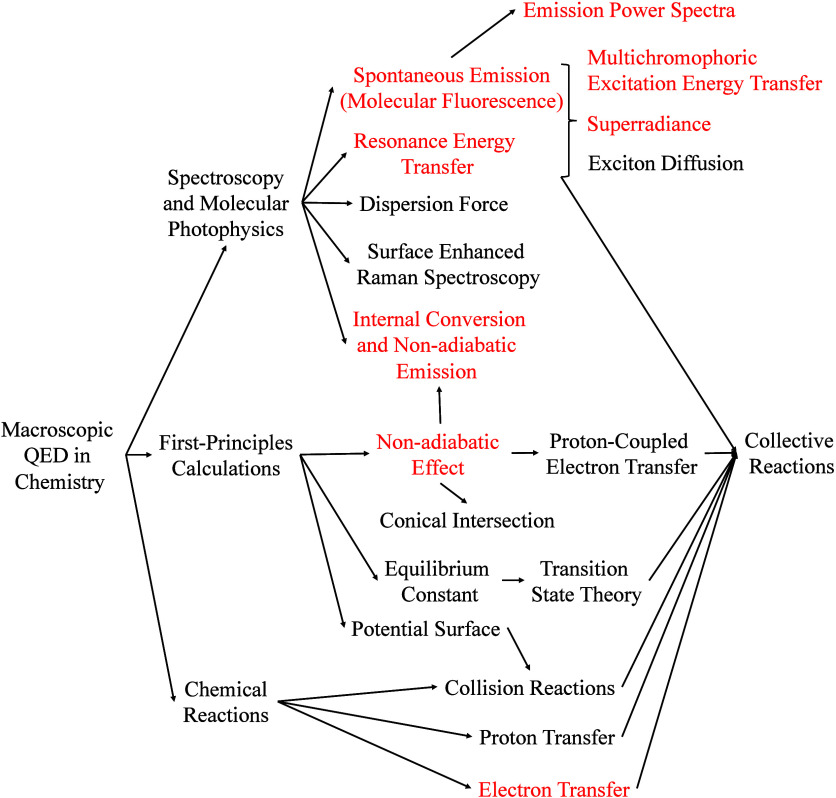
Prospective research directions of macroscopic QED in chemical sciences. The parts in red correspond to my research directions in recent years.

In recent years, macroscopic QED has been applied to the fields of quantum optics and nanophotonics,^112,113^ but its integration with physical chemistry remains rare. From my point of view, the integration of macroscopic QED and physical chemistry can be divided into three main aspects, as shown in Figure 2. The first part is spectroscopy and molecular photophysics, the second is first-principles calculation, and the third is chemical reactions.

The first part focuses on the photophysical properties of molecules, such as spontaneous emission and resonance energy transfer. Although these topics have been extensively studied in macroscopic QED, they have mostly been explored within the regime of weak light–matter coupling in nonchiral media. It was not until a few years ago that resonance energy transfer in chiral media began to be studied in detail.^114−116^ It should be noted that the synergistic effects of spontaneous emission and resonance energy transfer are crucial.^117−121^ These effects can be applied to multichromophoric excitation energy transfer,^118,119^ superradiance,^66,122^ and exciton diffusion,^123^ and they also form the basis for studying the collective effects in chemical reactions. Without calculating the coupling and dissipation of molecular interactions, as demonstrated in our studies,^64,66^ it is impossible to determine how many molecules simultaneously participate in QED-driven chemical reactions. The second part is a highly novel but challenging direction. Although Rubio and collaborators have successfully established the cavity BO approximation^56^ and calculated molecular electronic structures and potential surfaces within the framework of cavity QED, macroscopic QED involves dispersion and energy dissipation, making it fundamentally different from cavity QED. The integration of macroscopic QED with first-principles calculations to explore the effects of electromagnetic vacuum fields on chemical systems poses a grand theoretical challenge. The third part addresses how macroscopic QED can be combined with classical chemical reaction theories to understand how the electromagnetic vacuum field influences chemical reactions, such as the emergence of new electron-transfer mechanisms driven by QED and the control of electron transfer rates through the dielectric environment.

Building on these three aspects, I will now introduce the progress my group has made in recent years, focusing on the following topics.

1. Molecular fluorescence
2. Resonance energy transfer
3. Electron transfer

I hope these topics serve as a starting point to inspire readers and illustrate that macroscopic QED is not only a highly useful theoretical tool for polariton and QED chemistry but also offers new perspectives for experimental design.

### Molecular Fluorescence

When a molecule is excited to its electronic excited state, it quickly relaxes to its vibrational ground state within the same electronic state. It then returns to the electronic ground state through a spontaneous emission process, releasing a photon with the corresponding energy. This phenomenon is called fluorescence. Molecular fluorescence in a dielectric environment has been studied for more than 50 years. It is well-known that the decay rate of molecular fluorescence oscillates according to the distance between a molecule and a metal surface. To explain the oscillatory behavior of fluorescence rates, Kuhn, Chance, and Silbey developed a series of theories based on classical electrodynamics which demonstrate how the molecular distance from a metal surface influences the fluorescence decay rate.^124,125^ This framework is also known as the Chance-Prock-Silbey theory.^126^

The decay rate of molecular fluorescence in the presence of dielectrics can also be derived using Fermi’s golden rule from quantum electrodynamics.^102^ In other words, when molecules weakly couple to the electromagnetic vacuum field, classical and quantum electrodynamics yield a consistent result:^108^ the population of the electronic excited state decays exponentially, and the fluorescence rate enhancement (Purcell factor) can be expressed as a single-point dyadic Green’s function as
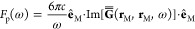
5which corresponds to the photonic local density of states. Here, a single-point dyadic Green’s function means that the positions **r** = **r**′ = **r**_M_ are for the two-point dyadic Green’s function  in eq 4, where **r**_M_ is the position of a molecule and **ê**_M_ is the unit vector of the molecular transition dipole moment. Thus, the mechanism of fluorescence can be understood as the molecular emission behavior being influenced by the local photonic density of states, which varies with different dielectric environments and affects the fluorescence rate.

The previous theoretical frameworks are limited to weak coupling regimes and cannot account for fluorescence behaviors when strong light–matter interactions occur. To answer this question, Wang, Scholes, and I established a unified theory of molecular fluorescence from weak to strong light–matter couplings within the framework of macroscopic QED.^127^ Our approach provides a unified framework that extends the applicability of classical theories by seamlessly integrating quantum electrodynamic effects, offering insights into fluorescence dynamics across nano-optics and polariton chemistry scenarios.

**Figure 3:**
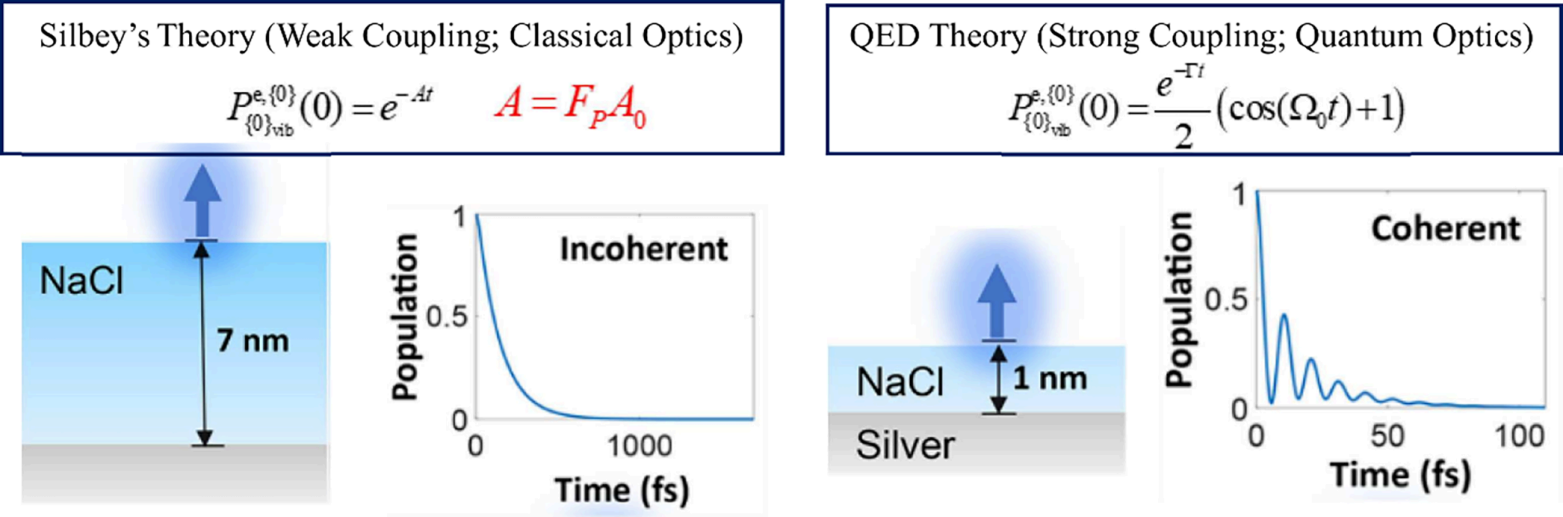
Based on macroscopic QED, my group developed a unified theory of molecular fluorescence that spans from weak to strong light–matter coupling regimes. In the weak coupling regime, where a molecule interacts with plasmon polaritons, the population dynamics reduce to those described by the Chance–Prock–Silbey theory. Adapted with permission from ref (128). (Copyright 2020, American Chemical Society.)

We started from the minimal coupling Hamiltonian under the electric dipole approximation, modeled a two-electronic-level molecule with multiple vibrational modes using the extended Wigner-Weisskopf wave function ansatz, and derived the following Volterra-type integro-differential equations as^127−129^
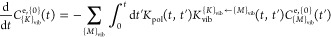
6where *C*_{*K*}_vib__^e,{0}^(*t*) stands for the coefficient for the electronically excited state of a molecule with the set of the quanta of all vibrational modes {*K*}_vib_ and a zero-polariton state {0} . The integro-differential equations are governed by the polaritonic kernel *K*_pol_(*t*, *t*′), which describes the dynamics resulting from photon-electronic state interactions and the vibrational kernel *K*_vib_^{*K*}_vib_ ←{*M*}_vib_^(*t*, *t*′), which describes molecular vibrational dynamics. Based on this theory, we explored the distance dependence of fluorescence rate enhancement and demonstrated the coherent-to-incoherent transition of molecular fluorescence for a chromophore above a silver surface,^128^ as shown in Figure 3. When the molecule is relatively far from the metal surface (Figure 3, left), it weakly couples with the plasmon polaritons, and the population of the electronic excited state decays exponentially, consistent with the part of the radiative rate in the Chance-Prock-Silbey theory. When the molecule is closer to the metal surface (Figure 3, right), it strongly couples with plasmon polaritons, forming an exciton–plasmon polariton, and the population of the electronic excited state exhibits Rabi oscillations.

The theory has several advantages:

(i) It is general for molecular fluorescence in dielectric environments with spatially dependent, frequency-dependent, or complex dielectric functions. In other words, the effects of materials (dielectric environment) are included in our theory.
(ii) The polariton memory kernel in eq 6 can be expressed in terms of single-point dyadic Green’s function, *K*_pol_(*t*, *t*′) = (2π)^−1^∫_0_^∞^ d*ω A*_0_(ω) *F*_p_(ω) *e*^–*i*(ω–ω_eg_) (*t*–*t*′)^, with the spontaneous emission rate in vacuum *A*_0_(ω) and the gap between two electronic states ω_eg_. The single-point dyadic Green’s function is inside the Purcell factor *F*_p_(ω) (Recall eq 5). In other words, one can calculate the polariton memory kernel using computational electrodynamics packages.
(iii) In the limit of high vibrational frequency, we demonstrated that the frequency of Rabi oscillations in population dynamics is associated with the Franck–Condon factor (Franck–Condon-Rabi oscillations).^127^
(iv) Under specific conditions, we derived a parameter-free formula to estimate exciton-polariton coupling.^64,127^ The estimated coupling values (78 meV) agree well with recent experimental results (80–95 meV) reported by the Cavendish Laboratory.^130^

In addition to molecular fluorescence, my group also established the theory of molecular emission power spectra based on macroscopic QED and demonstrated the interplay between exciton-photon and electron–phonon interactions.^67,129^ The intensity of the emission power spectrum can be decomposed into a line shape function and an electromagnetic environment factor.^129^ The former describes the fluorescence behavior of the molecule in a complex dielectric environment (quantum dynamics of the molecule), while the latter considers the effects of photon propagation from the molecule to the detector.^67^ The theory clearly captures physical phenomena ranging from the Franck–Condon pattern (weak coupling) to the polariton pattern (strong coupling) without free parameters, indicating that this framework can be applied to study the quantum dynamics of molecular polaritons.^131^

### Resonance Energy Transfer

Resonance energy transfer refers to the process where energy is transferred from an excited donor molecule to a ground-state acceptor molecule. This phenomenon has been extensively studied in physical chemistry for over 90 years.^132,133^ The theoretical foundation of resonance energy transfer can be traced back to Förster′s theory,^134^ formulated in 1948. Förster′s theory states that the energy transfer rate is proportional to the inverse sixth power of the intermolecular distance and the spectral overlap *J*, which is determined by the normalized fluorescence intensity spectrum of the donor *I*(ν̅) and molar absorption coefficient of the acceptor ε(ν̅), with ν̅ representing the wavenumber. It is important to note that Förster’s theory relies on electrostatic interactions and cannot describe retardation or radiative effects caused by electrodynamics. To address these limitations, McLone and Power first calculated the matrix element for energy transfer valid for all separations using molecular QED in 1964,^135^ with the inverse square dependence in the far-zone noted by Fermi in 1932.^136^ Furthermore, inspired by Power and Thirunamachandran, Andrews also utilized molecular QED to establish a unified theory of resonance energy transfer encompassing both radiative and nonradiative processes.^77,78^ The molecular QED framework demonstrates that the nonradiative process corresponds to Förster’s theory. Moreover, it extends Förster’s framework by incorporating retardation and radiation effects, thereby providing a more comprehensive description of long-range resonance energy transfer.

Förster theory has been widely applied in various fields, including photosynthesis (to describe energy transfer between pigments),^137^ organic optoelectronic materials (to optimize exciton transfer),^138^ and biological imaging (as a tool for distance measurement at the nanoscale).^139^ Several experiments have demonstrated that plasmonic materials can enhance the efficiency of resonance energy transfer,^140−142^ suggesting their potential to improve the performance of light-harvesting devices, particularly in plasmonic-enhanced photovoltaics and photocatalysis. However, an important question remains: can Förster theory or the molecular QED-based theory adequately explain the relationship between plasmonic materials and molecular resonance energy transfer? The answer appears to be negative. Plasmonic materials correspond to dispersive and absorptive media with a negative real part of the dielectric function, while molecular QED primarily applies to molecules in vacuum conditions.

Within the framework of macroscopic QED, the effects of electrodynamics and arbitrary inhomogeneous, dispersive, and absorptive media can be rigorously incorporated into molecular interactions. Ding, Schatz, and I combined macroscopic QED with the T-matrix formalism to extend the Förster theory.^143,144^ This extension accounts for the effects of complex electromagnetic environments, which were not previously considered. We derived an expression for the energy transfer rate in a Förster-type form in Gaussian unit:
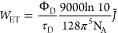
7where the donor lifetime τ_D_, the quantum yield Φ_D_, and the Avogadro number N_A_ are the same as those in Förster theory, and *J̃* is the generalized spectral overlap, expressed as
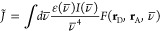
8Note that eq 8 is expressed in terms of wavenumber (not angular frequency) in Gaussian units. Equation 8 incorporates the donor emission spectrum, the acceptor absorption spectrum, and the electromagnetic coupling factor *F*(**r**_D_, **r**_A_, ν̅), where **r**_D_ and **r**_A_ represent the positions of the donor and acceptor, respectively. The first two factors are identical to those in Förster theory. It is important to note that the effect of dielectric environments is embedded in the electromagnetic coupling factor,
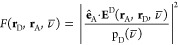
9The electromagnetic coupling factor offers a clear physical interpretation: resonance energy transfer is governed by the alignment between the electric field **E**^D^(**r**_A_, **r**_D_, ν̅) generated by the donor transition dipole moment p_D_(ν̅) at the acceptor’s position and the direction of the acceptor transition dipole **ê**_A_. The core mechanism of plasmon-enhanced energy transfer lies in amplifying the electromagnetic coupling factor via the donor-generated electric field, which is contributed from plasmon polaritons.

**Figure 4:**
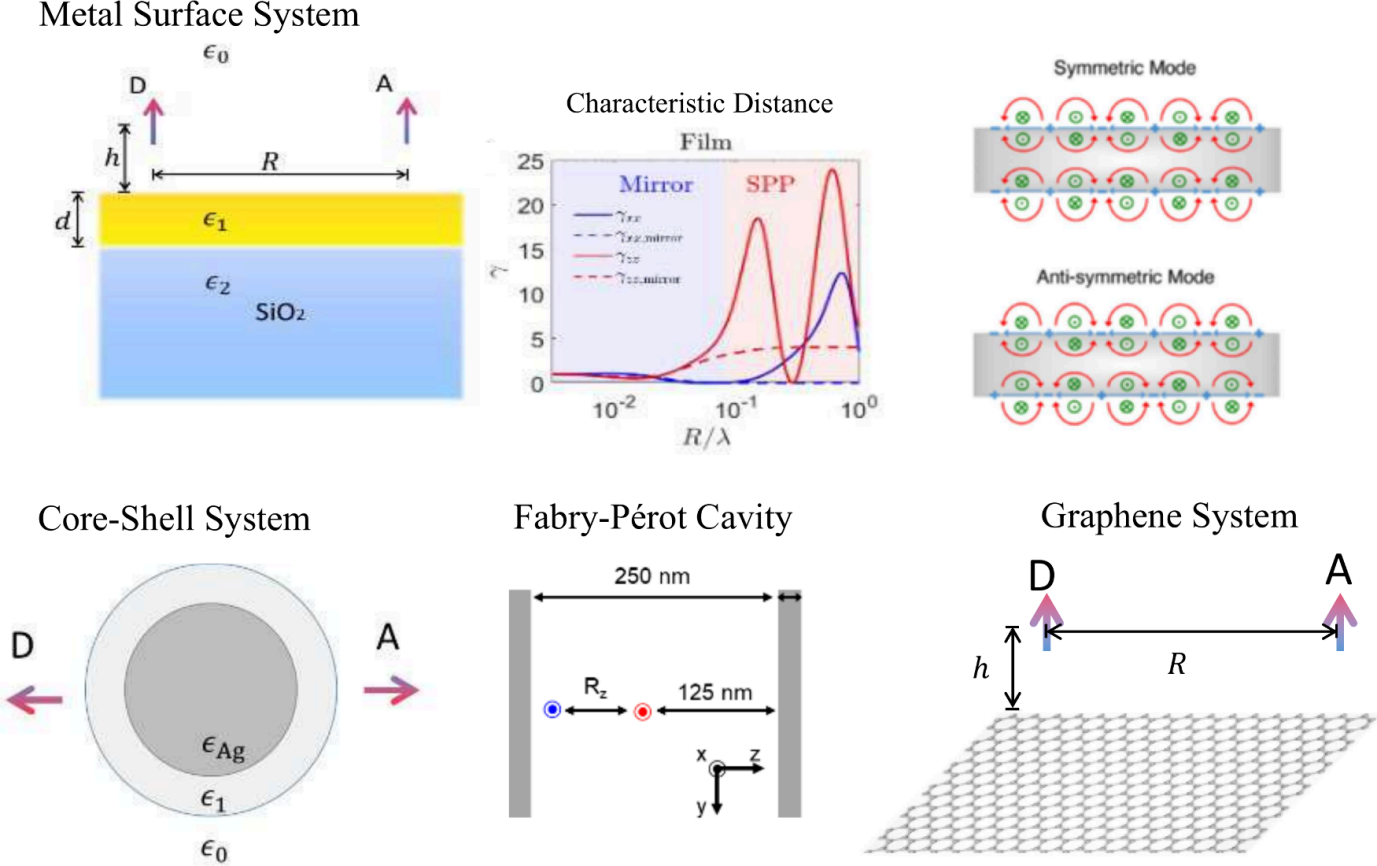
The theory of PCRET can be applied to describe resonance energy transfer in various systems, including molecules above a metal surface, around a core–shell particle, inside an FP cavity, and above a graphene layer. In other words, the macroscopic QED framework allows us to investigate molecules coupled with polaritons and cavity photons, providing guidance for designing materials to enhance resonance energy transfer. The panel of the metal surface system is adapted with permission from ref (145) (Copyright 2018, American Chemical Society),; the panel of the core–shell system is adapted with permission from ref (146) (Copyright 2020, American Chemical Society); and the panel of the FP cavity is adapted with permission from ref (147) (Copyright 2021, American Chemical Society).

**Figure 5:**
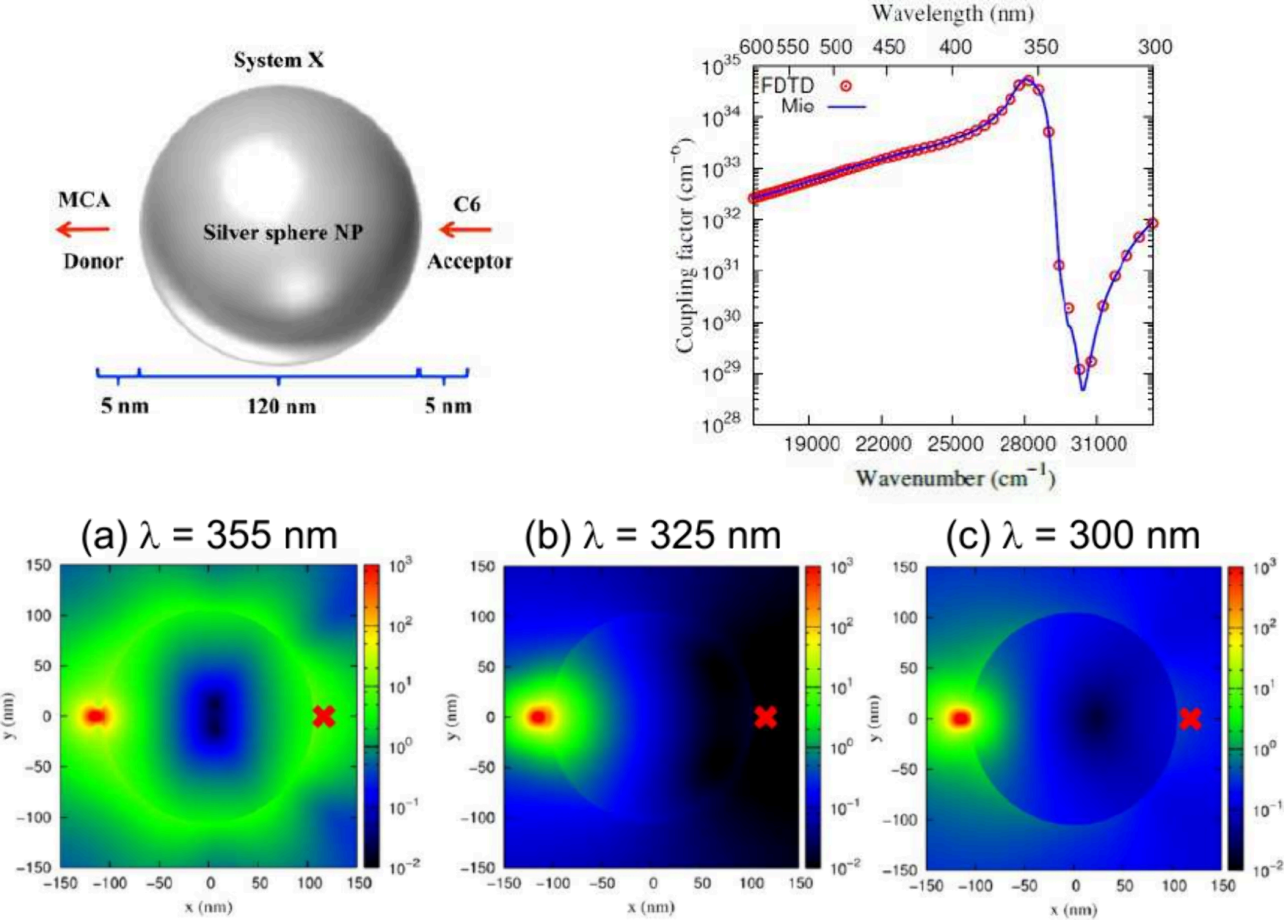
Theory of PCRET provides a visualization of resonance energy transfer pathways. The colors in (a), (b), and (c) correspond to the intensities of the donor-generated electric field, which represent the energy transfer pathways. (a), (b), and (c) show that these pathways vary dramatically with frequency, indicating different underlying energy transfer mechanisms. When λ = 355 nm, energy transfer propagates around the surface of the silver nanoparticle, indicating that the underlying mechanism involves energy transfer governed by plasmon polaritons. (b) When λ = 325 nm, energy transfer is blocked by the silver nanoparticle, indicating that the underlying mechanism involves energy transfer absorbed by plasmon polaritons. When λ = 300 nm, energy transfer weakly penetrates through the silver nanoparticle, indicating the underlying mechanism is energy transfer governed by polarization. Adapted with permission from ref (143) (Copyright 2017, American Chemical Society).

The theory offers several advantages:

(i) The theory provides a general framework for describing resonance energy transfer in dielectric media with “arbitrary spatially dependent, frequency-dependent, or complex dielectric functions. Although the theory was previously referred to as the theory of plasmon-coupled resonance energy transfer (PCRET), but I would like to emphasize that this theory is not limited to molecules near plasmonic materials.^144−146^ It can also be applied to describe resonance energy transfer in molecules within FP cavities^147^ or on 2D materials, as illustrated in Figure 4, showcasing such applications.
(ii) The donor-generated electric field at the acceptor can be expressed through a two-point dyadic Green’s function, e.g.,  = in Gaussian units, which can be efficiently computed using electrodynamics simulation tools.^148^
(iii) The two-point dyadic Green’s function corresponds to the propagation of electromagnetic waves (or photons), allowing visualization of resonance energy transfer pathways. Figure 5 demonstrates how these pathways vary dramatically with frequency, reflecting different underlying energy transfer mechanisms.^143^
(iv) In vacuum, the two-point dyadic Green’s function simplifies to the vacuum case, and the PCRET formalism converges to Andrews′ result. This unification demonstrates that the theory integrates both Andrews′ and Förster’s studies.^143^

These advantages illustrate how macroscopic QED not only advances our understanding of QED effects in chemical systems but also enriches our appreciation of classical physical chemistry theories.

Despite its strengths, the PCRET theory has several limitations. First, it is limited to energy transfer between a single donor–acceptor pair and cannot describe energy transfer within a group of molecules. This collective energy transfer is crucial for exciton diffusion^149,150^ and photosynthesis.^151−156^ To address this limitation, my group developed a macroscopic QED-based theory of multichromophoric excitation energy transfer,^118−121^ incorporating both molecular fluorescence and resonance energy transfer. For molecules weakly coupled to plasmon polaritons, Ding and my group used the kinetic approach to calculate the exciton diffusion coefficient on plasmonic materials,^123^ finding an enhancement of up to 3 orders of magnitude. Second, PCRET theory is based on the dipole approximation, making it unsuitable for describing energy transfer in large polymers or biomolecules. Previous studies by Scholes^157^ and Hsu^158^ utilized transition density methods to calculate resonance energy transfer between large biomolecules. However, this approach does not account for retardation, radiation, or complex medium effects. To address these challenges, my group developed a unified theory of radiative and nonradiative resonance energy transfer, based on transition current density within the macroscopic QED framework.^159^ This theory can be reduced to both the PCRET and transition density methods in specific conditions, but its computational complexity may restrict practical applications. Finally, PCRET assumes weak coupling between molecules and the electromagnetic vacuum field, limiting its ability to describe collective effects or superradiance.^66,122^

### Electron Transfer

The control of chemical reactions is one of the most important issues in the field of chemistry.^160−164^ Among chemical reactions, electron transfer is the most fundamental and widely applicable, playing a critical role in organic, inorganic, and biochemical processes.^165−168^ Traditionally, the rate of electron transfer can be estimated using Marcus theory.^160,169^ According to Marcus theory, electron transfer mechanisms can be divided into two regions: the normal region and the inverted region. In the inverted region, Marcus theory predicts that the lower the energy of the product, the slower the electron transfer rate, which contradicts conventional chemical intuition. This groundbreaking discovery earned Marcus the Nobel Prize.

Whether electromagnetic vacuum fields can significantly influence chemical reaction rates is one of the core questions in QED chemistry. Within the framework of cavity QED, Semenov and Nitzan considered electron transfer inside a cavity and developed a single-photonic-mode electron transfer theory in 2019.^170^ The Semenov-Nitzan theory predicted that under strong coupling between a single photonic mode and a molecule, the single photonic mode can greatly enhance the electron transfer rate and eliminate the inverted region. Their pioneering work inspired me to ask the following question: Can electromagnetic vacuum fields formed by infinite photonic modes in vacuum or homogeneous media also significantly affect the electron transfer process? Previous studies suggest that infinite photonic modes weakly couple with molecules, leading to negligible QED effects.^30^ In other words, electromagnetic vacuum fields formed by infinite photonic modes are generally thought to have little or no impact on electron transfer. However, does this intuition hold true under all conditions?

**Figure 6:**
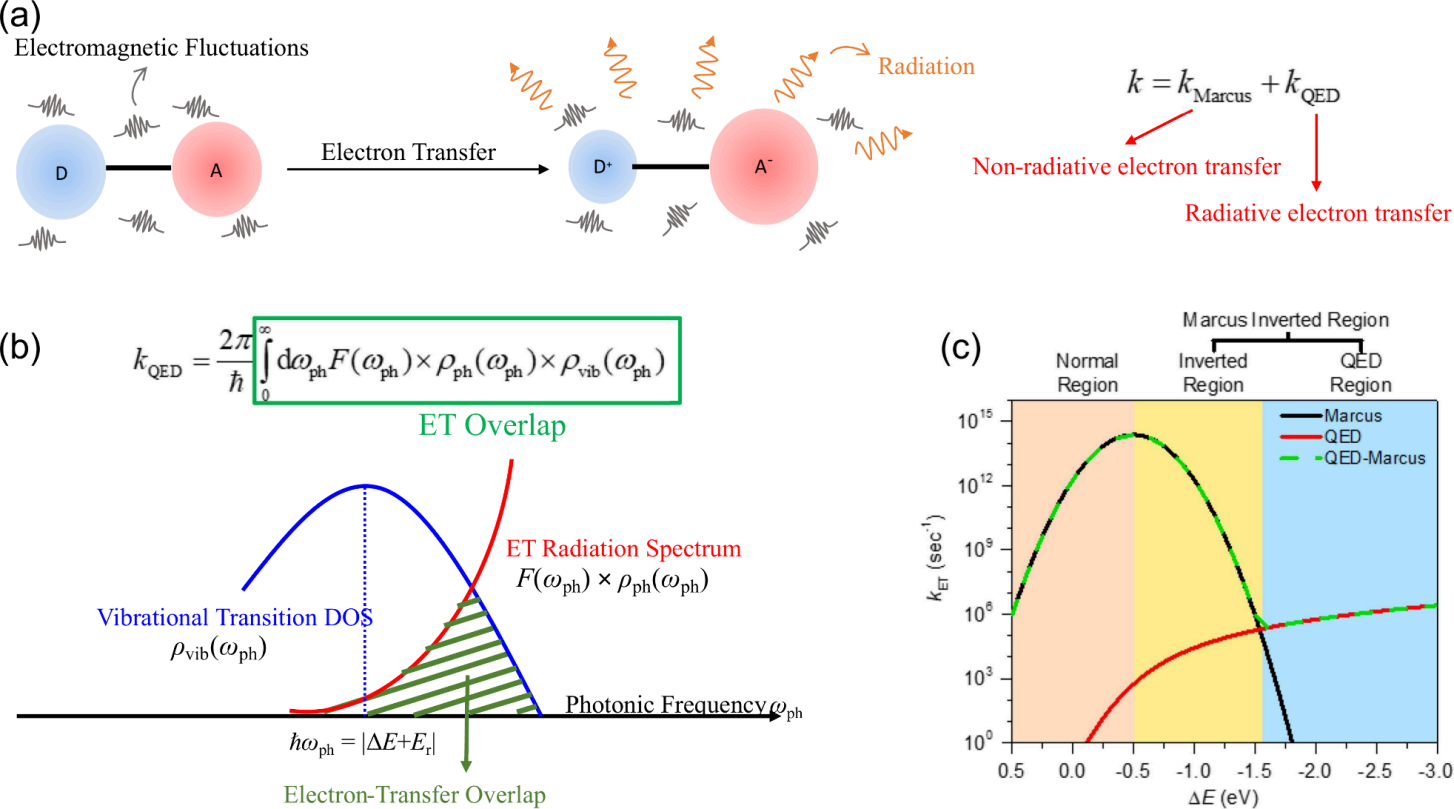
Based on molecular QED, my group has developed a unified theory for nonradiative and radiative electron transfer processes. (a) The total electron transfer rate is separated into the Marcus electron transfer rate *k*_Marcus_ and the QED-driven electron transfer rate *k*_QED_. (b) The mechanism of the QED electron transfer can be understood through the concept of electron transfer overlap, an integral involving the photonic density of states (DOS) ρ_ph_(ω_ph_), vibrational transition DOS ρ_vib_(ω_ph_), and the light–matter coupling factor F(ω_ph_). (c) In the QED framework, the classical Marcus inverted region can be further separated into two regions: the inverted region and the QED region. Adapted with permission from ref (171) (Copyright 2022, American Chemical Society).

To answer this question, Wei and I investigated electron transfer processes under weak coupling between molecules and infinite photonic modes in the absence of a cavity. Based on molecular QED, we started from the minimal coupling Hamiltonian (spinless) within the electric dipole approximation and extended Marcus electron transfer theory to a version of cavity-free quantum-electrodynamic electron transfer (QED-ET).^171^ Based on the Fermi’s golden rule, the total electron transfer rate in molecular QED is expressed as
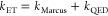
10where *k*_Marcus_ stands for the nonradiative electron transfer rate, which is identical to the Marcus rate,^169^ and *k*_QED_ represents the radiative electron transfer driven by QED effects, as shown in Figure 6a. By explicitly incorporating electromagnetic vacuum fields, this unified framework recovers radiative contributions to electron transfer, which are absent in traditional Marcus theory that considers only Coulomb interactions. To make it more convenient for readers, I will refer to the QED-ET theory based on molecular QED as the vacuum QED-ET theory (as molecular QED primarily addresses the interactions between molecules and photonic modes in a vacuum).

Furthermore, the QED electron transfer can be understood through the concept of electron transfer overlap, an integral involving the photonic density of states (DOS) ρ_ph_(ω_ph_), vibrational transition DOS ρ_vib_(ω_ph_), and the light–matter coupling factor F(ω_ph_),
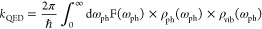
11where ω_ph_ is the photonic frequency, as shown in Figure 6b. In free space, ρ_ph_(ω_ph_) = ω_ph_^2^/π^2^c^3^ and *F*(ω_ph_) = |**μ**_DA,*D*^+^*A*^–^_|^2^ℏω_ph_/6ε_0_, where **μ**_DA,*D*^+^*A*^–^_ denotes the transition dipole of two electron-transfer states.

Based on eqs 10 and 11, we can plot the relationship between the energy gap *ΔE* and *k*_ET_, as shown in Figure 6c. According to the trend of electron transfer rates varying with the energy gap, the mechanism of electron transfer in Figure 6c can be divided into three regions: the normal region, the inverted region, and the QED region (or the highly inverted region). In the QED region, the electron transfer rate increases with the energy gap, which contradicts the Marcus theory. However, this phenomenon is not unexpected, as higher-energy electronic states possess greater kinetic energy, leading to stronger radiative capabilities (enhanced radiative transitions driven by electrodynamic effects). Therefore, the electron transfer process does not rely on Coulomb interactions between charges but instead proceeds through a spontaneous emission-like mechanism. Note that the QED region specifically arises due to QED effects from electromagnetic vacuum fields induced by infinite photonic modes and is independent of traditional Marcus theory.

The vacuum QED-ET theory provides two crucial insights. First, as long as the electronic state of a molecule possesses high energy, radiative electron transfer processes become almost unavoidable, offering a possible mechanism for the experimentally observed disappearance of the inverted region.^172−174^ Second, quantum electrodynamic effects can significantly influence electron transfer rates even under weak coupling between electromagnetic vacuum fields and molecules, addressing the question I raised earlier.

**Figure 7:**
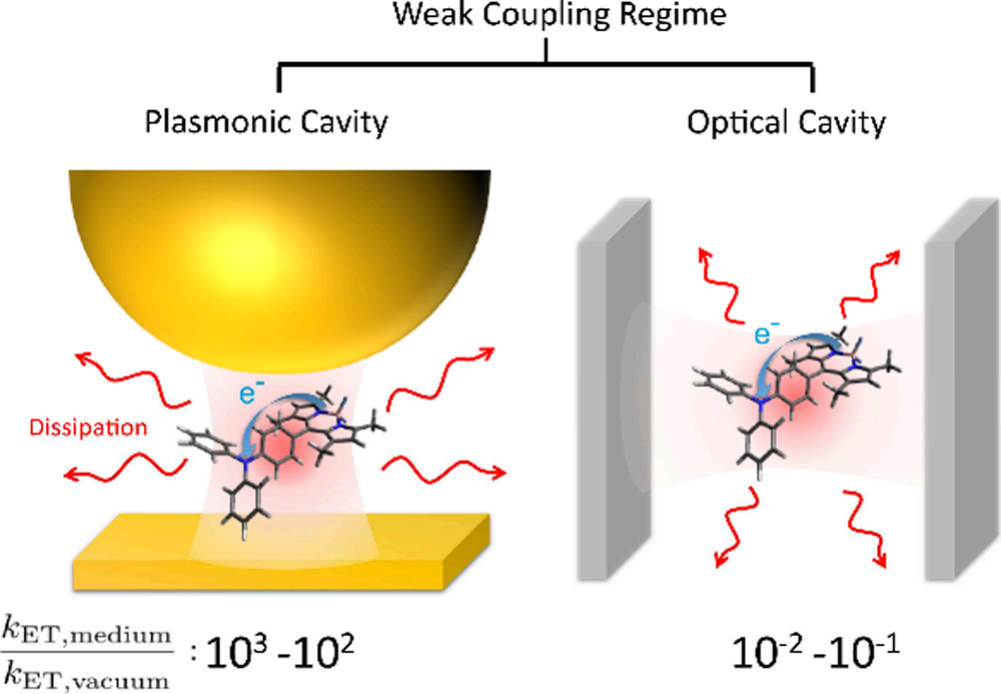
Enhancement of electron transfer rates for molecules in different dielectric environments. The left corresponds to a molecule coupled to plasmon polaritons, while the right corresponds to a molecule coupled to cavity photons. Based on the macroscopic QED-ET theory, the enhancements are 10^2^–10^3^ times for the plasmonic cavity and 10^–2^–10^–1^ times for the FP cavity. Reprinted with permission from ref (176) (Copyright 2024, American Chemical Society).

To investigate the effects of different dielectric environments on electron transfer rates, Wei and I developed the concept of the polaritonic Huang–Rhys factor,^175^ which can be used to quantitatively describe the effects of (i) light–matter coupling caused by permanent dipoles and (ii) dipole self-energy. Furthermore, by using the polaritonic Huang–Rhys factor, we established a macroscopic QED version of the electron transfer theory. Hereafter, this will be referred to as the macroscopic QED-ET theory for brevity. Under this theoretical framework, we investigated two systems (dielectric environments): an electron transfer system placed in a plasmonic cavity, illustrated in the left panel of Figure 7, and an electron transfer system placed in an FP cavity, illustrated in the right panel of Figure 7. The former corresponds to a molecule coupled to plasmon polaritons, while the latter corresponds to a molecule coupled to cavity photons. We ensured both systems operated under weak coupling conditions by selecting appropriate physical conditions. After calculating the electron transfer rates using the macroscopic QED-ET theory, we found that the enhancements were 10^2^ – 10^3^ times for the plasmonic cavity and 10^–2^ – 10^–1^ times for the FP cavity. This indicates that even under weak coupling between molecules and electromagnetic vacuum fields, material design can yield up to 5 orders of magnitude difference in electron transfer rates, offering new insights into strategies for controlling chemical reactions.

**Figure 8:**
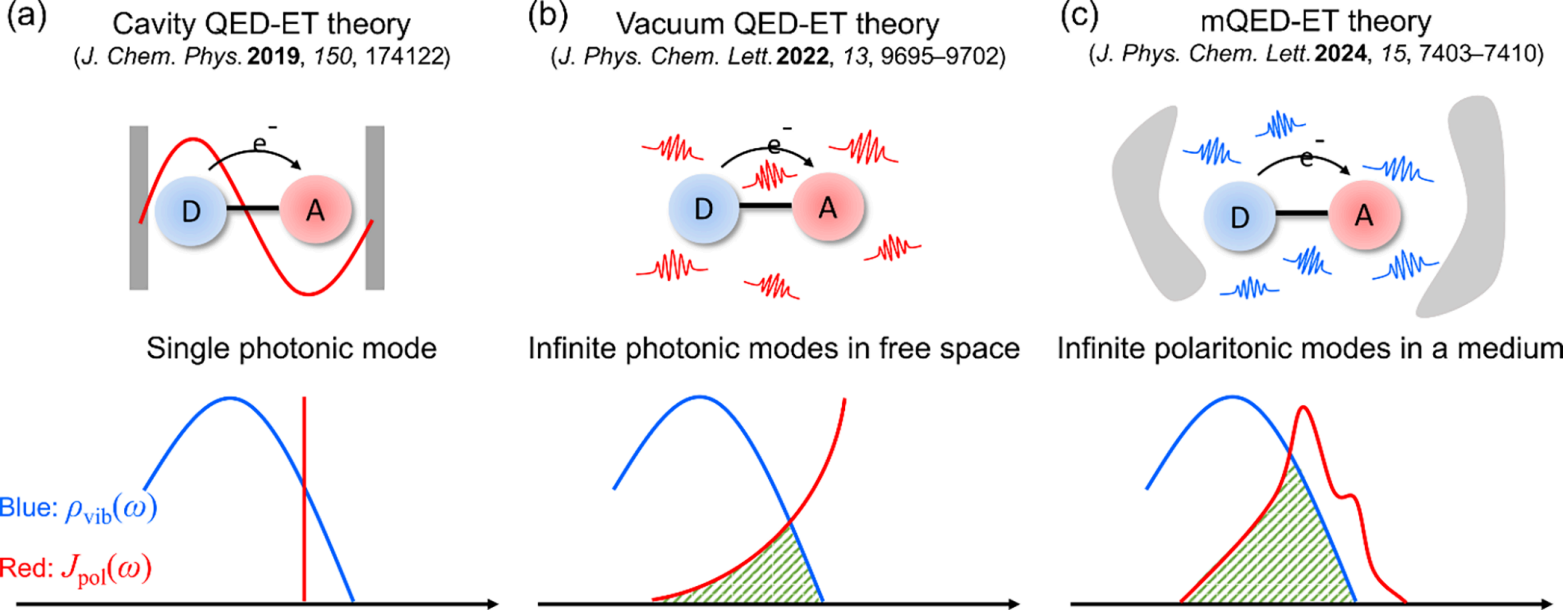
(a–c) Vibrational transition DOS, photonic/polaritonic spectral density, and electron-transfer overlap under different QED frameworks. Adapted with permission from ref (176) (Copyright 2024, American Chemical Society).

In addition, based on macroscopic QED, we derived a generalized expression for *k*_QED–ET_ as follows:
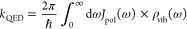
12where *J*_pol_(ω) is related to the molecular electron transfer transition dipole and the local polaritonic DOS. When the polaritonic Huang–Rhys factor is small, eq 12 can be expressed in terms of the single-point dyadic Green’s function as
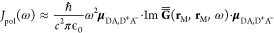
13In the case of free space, our previous study has shown that eq 13 can be reduced to eq 11, i.e., *J*_pol_(ω) = F(ω_ph_) ρ_ph_(ω_ph_). This demonstrates that the macroscopic QED-ET theory is consistent with the vacuum QED-ET theory, and the vacuum QED-ET theory can be considered a special case of the macroscopic QED-ET theory. The significance of eq 12 lies in its compatibility with describing electron transfer rates under various QED frameworks, including cavity QED, molecular QED, and macroscopic QED. To help readers understand the concept of electron transfer overlap, Figure 8a, b, and c correspond to electron transfer overlaps under three different QED frameworks: cavity QED, molecular QED, and macroscopic QED, respectively.

In this Perspective, I first reviewed the fundamental role of light–matter interactions in chemistry, focusing on both traditional areas, such as molecular fluorescence and dispersion forces, and emerging fields, such as polariton chemistry. This overview underscores the need for an in-depth exploration of QED effects in chemistry. However, existing frameworks, such as cavity QED and single-photonic-mode models, hinder accurate prediction of molecular interactions with electromagnetic vacuum fields. To address these limitations, I presented macroscopic QED–developed by Welsch, Scheel, Buhmann et al.–to the physical chemistry community as a framework that accounts for the effects of infinite photonic modes and dielectric environments.

In the section on macroscopic quantum electrodynamics, I aim to provide readers with a comprehensive understanding of QED classifications by introducing different types of QED, including the core models of cavity QED, as shown in Figure 1. I then reviewed the development of macroscopic QED, emphasizing its rigor in addressing complex dielectric environments. To clarify the distinctions between macroscopic QED and traditional QED frameworks for polariton chemistry researchers, I used the example of the electric field operator to illustrate how macroscopic QED accounts for absorption, dispersion, and boundary effects in dielectric materials.

In the section on macroscopic QED in chemistry, I outline the research applications of macroscopic QED by categorizing them into three main directions: spectroscopy and molecular photophysics, first-principles calculations, and chemical reactions, as shown in Figure 2. To illustrate how macroscopic QED can be applied in physical chemistry, I used my research to present three representative areas: molecular fluorescence, resonance energy transfer, and electron transfer.

In the subsection on molecular fluorescence, I introduced the generalized theory of molecular fluorescence developed by Schatz, Ding, and me. Within the framework of macroscopic QED, our theory not only encompasses the key results of the Chance-Prock-Silbey theory (a foundational milestone in nano-optics) but also captures the Rabi oscillations of light–matter hybrid states (one of the most important features in polariton chemistry and quantum optics) in a unified theoretical framework without adjustable parameters, as shown in Figure 3. I also briefly introduced the theory of molecular emission power spectra. This theory investigates the quantum dynamics arising from the interplay between exciton-photon and electron–phonon interactions and quantitatively calculates the emission intensity of molecules in dielectric materials, which is critical for applications in biomedical sensing and diagnostics.

In the subsection on resonance energy transfer, I introduced the generalized theory of resonance energy transfer developed by my group. Within the framework of macroscopic QED, our theory not only provides a unified description of both radiative and nonradiative resonance energy transfer but also encompasses the iconic results of Andrews and Förster. Next, I demonstrated that our theory describes resonance energy transfer of molecules in various dielectric environments (including plasmon polaritons and cavity photons, Figure 4) and visualizes the energy transfer pathways within the medium (Figure 5).

In the subsection on electron transfer, I introduced the generalized theory of electron transfer developed by my group. Figure 6 shows that the molecular QED-ET theory provides a unified description of Marcus theory and radiative electron transfer. Figure 7 demonstrates that the macroscopic QED-ET theory describes electron transfer coupled with electromagnetic vacuum fields in a plasmonic cavity (plasmon polaritons) and in an FP cavity (cavity photons). Finally, our theory clearly shows that radiative electron transfer in both cavity and free-space conditions, encompassing the results of cavity QED and molecular QED, can be described in a unified theoretical framework (macroscopic QED), as shown in Figure 8.

Beyond the three representative research areas introduced in this perspective, my group has also applied macroscopic QED to the study of nonadiabatic QED effects,^177^ internal conversion,^178^ collective effects,^65^ and superradiance.^66,122^ Space limitations preclude a detailed discussion of these studies here. In addition to my group, Feist and García-Vidal et al. developed few-mode quantization methods for single emitter and multiple emitters,^179,180^ which bridged macroscopic QED and cavity QED. Flick et al. combined the first-principles method and macroscopic QED to investigate linear absorption spectra.^181^ Here, I would like to emphasize that accurately describing intermolecular interactions is crucial for understanding QED-driven collective effects in chemical reactions. In our previous research, we quantitatively demonstrated that, even under weak coupling between molecules and electromagnetic vacuum fields, counter-rotating interactions^120^ and direct intermolecular interactions^121^ cannot be neglected in the multipolar gauge (or dipole gauge). In other words, using single-photonic-mode models to describe QED-induced collective effects for closely spaced molecules may lead to misinterpretations of experimental phenomena. Furthermore, chemical reactions with vibrational strong coupling are performed in solution within a cavity. How to capture the complicated molecular interactions between molecules and solvent (or medium) is a critical issue in polariton chemistry. To address this issue, Schäfer et al. proposed a full macroscopic QED embedding approach and a quantized embedding radiation-reaction approach.^111,182^ It should be noted that, under strong light–matter interactions, nonadiabatic QED effects can emerge in electron–nucleus-photon systems. In view of this, we developed a generalized Born-Huang expansion to further understand QED-induced nonadiabatic effects.^177^

Although macroscopic QED has many advantages, its complex mathematical language (such as bosonic vector fields and dyadic Green’s functions) and the challenges of electrodynamic calculations often make it inaccessible to outsiders, thereby limiting its application in physical chemistry. This Perspective aims to provide a comprehensive and conceptual introduction to macroscopic QED, with the goal of inspiring future research, fostering a deeper understanding of QED effects in chemistry, and advancing the fundamental principles and applications of QED chemistry.
